# Expression of ATF6 as a marker of pre-cancerous atypical change in ulcerative colitis-associated colorectal cancer: a potential role in the management of dysplasia

**DOI:** 10.1007/s00535-017-1387-1

**Published:** 2017-09-07

**Authors:** Marie Hanaoka, Toshiaki Ishikawa, Megumi Ishiguro, Michiyo Tokura, Shinichi Yamauchi, Akifumi Kikuchi, Hiroyuki Uetake, Masamichi Yasuno, Tatsuyuki Kawano

**Affiliations:** 1 Department of Gastrointestinal Surgery, Graduate School, Tokyo Medical and Dental University, 1-5-45 Yushima, Bunkyo-ku, Tokyo, Japan; 2 Department of Specialized Surgeries, Graduate School, Tokyo Medical and Dental University, 1-5-45 Yushima, Bunkyo-ku, Tokyo, Japan; 3 Department of Translational Oncology, Graduate School, Tokyo Medical and Dental University, 1-5-45 Yushima, Bunkyo-ku, Tokyo, Japan

**Keywords:** ATF6, Unfolded protein response, Ulcerative colitis, Dysplasia, Colorectal cancer

## Abstract

**Background:**

Diagnosis of low-grade dysplasia (LGD) is important in the management of ulcerative colitis (UC), but it is often difficult to distinguish LGD from inflammatory regenerative epithelium. The unfolded protein response (UPR) is activated in inflammatory bowel disease and malignancies. We aimed to identify a UPR-related gene that is involved in the development of non-UC and UC-associated colorectal cancer (CRC), and to investigate whether the target gene is useful for the diagnosis of LGD.

**Methods:**

Using our microarray gene expression database of 152 CRCs, we identified activating transcription factor 6 (ATF6) as a target gene. Immunohistochemistry (IHC) of ATF6 were analyzed in 137 surgically resected CRCs, 95 endoscopically resected adenomas and pTis cancers, and 136 samples from 51 UC patients (93 colitis without neoplasia, 31 dysplasia, and 12 UC-associated CRC). The diagnostic accuracy of ATF6 and p53 as markers of LGD was assessed.

**Results:**

ATF6 expression was detectable in all CRCs but not in normal colonic mucosa, was elevated with increase in cellular atypia (adenoma with moderate atypia < severe atypia < pTis CRC, *p* < 0.001), and higher in dysplasia and CRC than in non-neoplastic colitis (*p* < 0.001). Notably, the difference between colitis and LGD was significant. Compared to p53-IHC, ATF6-IHC had better diagnostic accuracy for distinguishing LGD from background inflammatory mucosa (sensitivity 70.8 vs. 16.7%, specificity 78.5 vs.71.0%, respectively).

**Conclusions:**

ATF6 was expressed in lesions undergoing pre-cancerous atypical change in both non-UC and UC-associated CRC and may be used to distinguish LGD from inflammatory regenerative epithelium in UC patients.

**Electronic supplementary material:**

The online version of this article (doi:10.1007/s00535-017-1387-1) contains supplementary material, which is available to authorized users.

## Introduction

The incidence of ulcerative colitis (UC) is significantly increasing around the world [1]. It is well known that long-standing UC leads to colorectal cancer (CRC), and is often a threat to patients’ lives. The risk of developing UC-associated CRC increases 0.5–1% per year for patients with disease duration in excess of 8–10 years [2]. In addition, the prognosis of CRC is generally poorer in UC patients than in non-UC patients [3]. Therefore, surveillance colonoscopy is recommended for the detection of neoplasms in patients with UC. Early detection of UC-associated CRC is essential for successful management of long-standing UC [3]. However, endoscopic and histologic detection of dysplasia (i.e., precancerous lesions) is often difficult due to the presence of inflammatory change and subsequent regenerative change of the colonic mucosa [2, 4].

For early detection of dysplasia, it is important to understand the mechanism of CRC development in UC patients. While chronic inflammation of colonic mucosa is understood to be the cause of UC-associated CRC [5], the genetic details of UC-associated CRC pathogenesis are not fully understood [6]. p53 mutation has been reported to occur early in the development of UC-associated CRC [2, 6]. Immunohistochemistry (IHC) of p53 is therefore often used for the diagnosis of neoplastic lesions in UC. However, the usefulness of p53-IHC is still controversial. Detection of promising biomarkers for diagnosis of dysplasia, especially for diagnosis of low-grade dysplasia, could impact the clinical management of cancer risk in UC patients [2].

Recently, the unfolded protein response (UPR) is reported to be associated with a variety of diseases, including neurodegenerative disorders, diabetes mellitus, metabolic syndrome [7], inflammatory bowel disease [8–11], and several types of malignancies [12–15]. Physiological and pathological conditions that perturb the endoplasmic reticulum (ER), such as inflammation, nutrient deprivation, hypoxia, oxidative stress, and infection lead to “ER stress”, which includes accumulation of unfolded of misfolded proteins, and eventually leads to cell apoptosis [12]. UPR is one of the protective mechanisms against ER stress; by activating the UPR, the cells under ER stress re-establish cellular homeostasis and avoid apoptosis [16].

The UPR signaling pathway has three canonical branches, each mediated by a different ER stress sensor [12, 13, 16]: (1) inositol-requiring kinase 1 (IRE1), which activates a transcriptional factor X-box binding protein-1 (XBP-1) and then UPR genes involved in protein folding and ER-associated degradation [17], (2) activating transcriptional factor 6 (ATF6), which induces XBP-1 mRNA, and enhances protein folding and maturation [13], and (3) protein kinase RNA-like endoplasmic reticulum kinase (PERK), which inhibits translation of several mRNAs through phosphorylating the α-subunit of translation initiation factor 2 (eIF2α) [12]. Cancer cells are generally considered to be under ER stress, because their rapid growth might lead to insufficient supply of glucose, hypoxia, and inadequate vascularization. Accordingly, they constitutively activate the UPR system in order to adapt to stressful environments [12, 13].

We therefore hypothesized that UPR might play some role in the development and progression of CRC, especially in the oncogenic pathway of UC-associated CRC. Chronic inflammation, a possible cause of UC-associated CRC, can induce ER stress and subsequent UPR. In this study, using microarray analysis, we identified ATF6, one of the UPR related genes, as a target gene. ATF6 is a basic leucine zipper transcription factor belonging to the cyclic-AMP-responsive-element-binding protein (CREB) and ATF family of transcription factors [18]. Upon ER stress, ATF6 induces the transcription of genes encoding ER chaperones and enzymes that facilitate protein folding and maturation [12, 13, 16]. From the results of a previously reported comprehensive analysis of ER stress and UPR in UC patients, strong activation of the IRE1 and ATF6 pathway was observed in UC colonic tissue [19]. ATF6 is reported to also be activated in several types of cancer [12–15]. However, the correlation between intratumoral ATF6 expression and the development of CRC in both non-UC and UC patients has not been reported.

The aim of this study is to investigate the relationship between ATF6 expression and development of CRC, especially UC-associated CRC, and to evaluate whether ATF6 detection can be used for earlier diagnosis of dysplasia in UC patients.

## Methods

### Identification of a target gene by analysis of microarray gene expression data

We have constructed a gene expression database from the results of oligonucleotide microarray analysis using 152 CRC primary tumors. The database included 27, 69, and 56 patients who underwent surgical resection for stage I, II, and III CRC, respectively, between 2002 and 2009 at Tokyo Medical and Dental University Medical Hospital.

Serial frozen sections were mounted on glass slides, and laser capture microdissection (LCM) was carried out. Total RNA was extracted from the LCM of cancer tissues and normal colorectal epithelia using an RNeasy micro kit (Qiagen, Hilden, Germany) according to the manufacturer’s protocol. The integrity of the total RNA was assessed using an Agilent 2100 BioAnalyzer (Agilent Technologies, Palo Alto, CA, USA), and then cRNA was prepared from 100 ng of total RNA using two-cycle target labeling and a control reagents kit (Affymetrix, Santa Clara, CA, USA). Hybridization and signal detection of the Human Genome U133 Plus 2.0 Array (Affymetrix) were carried out according to the manufacturer’s instructions.

In this study, the gene expression database was used to identify candidate genes. The Wilcoxon exact rank-sum test (*p* < 0.001) was used to select genes whose expression level in cancer tissue was >1.5 times higher than that in non-neoplastic tissue. A total of 4416 genes were identified as fulfilling this criterion. Subsequently, we searched these 4416 candidate genes to identify those genes whose expression have been previously reported to be associated with UPR [12]. Finally, ATF6, was uniquely identified as a target gene of interest.

### Patients and samples for IHC study

A total of 283 patients were included in the IHC study. We obtained specimens from three series of patients: (1) 137 consecutive patients with non-UC CRC who underwent surgical resection for CRC between January 2010 and December 2011 (19, 18, 39, 45, and 16 patients with stage 0, I, II, III, and IV CRC, respectively), (2) 95 consecutive patients with adenoma or pTis cancer who underwent endoscopically resection between January 2010 and December 2011 (48 adenomas and 47 Tis CRC), and (3) 51 consecutive patients with UC who underwent colectomy between January 2004 and December 2015.

The diagnosis from surgically resected UC specimens was made by specialized pathologists. The pathologic findings of UC samples were categorized into two groups: absence for neoplasia group and neoplasia group. Neoplasia was graded according to the revised Vienna classification [20] as low-grade dysplasia (LGD), high-grade dysplasia (HGD), and UC-associated CRC. Mucosa without neoplastic change was divided into active and inactive colitis using the Histological Activity Index reported by Gupta et al. [21] (Supplemental file 1).

The characteristics of UC patients are shown in Supplemental file 2. Of 51 subjects, 33 had no neoplastic lesions and 18 had neoplasia. A total of 136 lesions from the 51 UC patients were used for IHC study including 39 inactive colitis, 54 active colitis, 24 LGD, 7 HGD, and 12 UC-associated CRC lesions (Supplemental file 1). Samples of colitis from patients with or without neoplasia were selected, in principle, from both the right and left sides of the colon (Supplemental files 3, 4). Samples of neoplasia were selected from each neoplastic lesion (Supplemental file 4).

### IHC staining

IHC analysis was performed on 4-μm sections from formalin-fixed paraffin-embedded tissue blocks from each patient. We performed ATF6-IHC analysis on all samples in this study and p53-IHC analysis on the UC samples. All sections were scored by two investigators.

#### IHC staining of ATF6

A streptavidin–biotin method was used for ATF6 immunostaining. After deparaffinization and rehydration, antigen was retrieved from the tissues in pH 6.0 citrate buffer using microwave heating at 98 °C for 25 min. Sections were incubated sequentially in a solution of 3% hydrogen peroxide in 100% methanol for 15 min at room temperature to quench endogenous peroxidase activity; in a solution of rabbit polyclonal antibody against ATF6 (1:150; ab203119 Abcam, Cambridge, UK) for 15 min at room temperature and 16 h at 4 °C; labeled polymer [Histofine^®^ Simple Stain MAX PO (MULTI); Nichirei Bioscience, Tokyo, Japan] for 30 min at room temperature, and DAB (0.02% 3, 30-diaminobenzidine tetrahydrochloride; Nichirei Bioscience) for 10 min at room temperature for visualization. The slides were counterstained with 1% Mayer’s hematoxylin, after which they were dehydrated using a series of increasing alcohol concentrations, followed by xylene immersion and finally coverslipping.

**Fig. 1:**
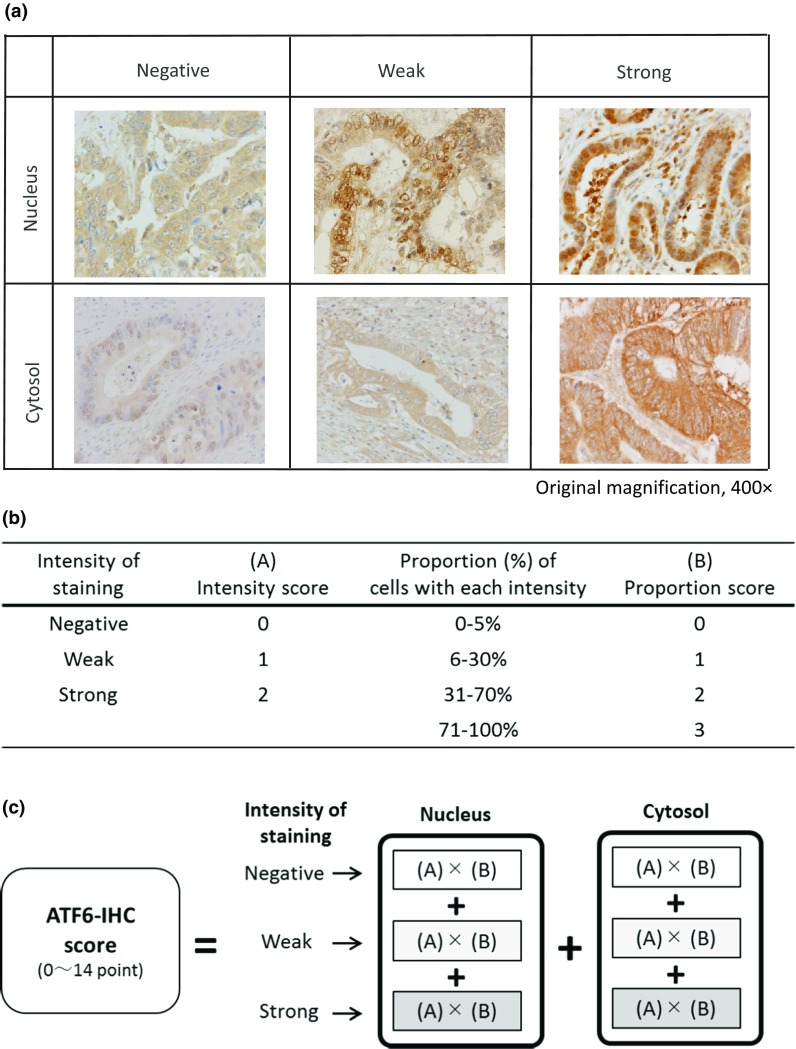
Evaluation of ATF6-IHC staining. **a** ATF6 immunostaining intensity in the nucleus and cytosol. **b** ATF6-IHC rating for intensity and proportion. **c** Final ATF6-IHC score

ATF6-IHC was evaluated using a modification of the method described by Krajewska et al. [22]. First, five fields (magnification 100×) were selected at the invasive front of the CRC samples, in areas with the most atypical histology in the adenoma and UC-dysplasia samples, and areas with the most marked inflammatory change in the colitis-without-neoplasia samples. The nucleus and cytosol were evaluated separately. Immunostaining intensity was graded as 0 (negative), 1 (weak) or 2 (strong) (Fig. 1a, b). The number of cells with each intensity of staining was counted independently and totaled in the 5 fields. Scores corresponding to the proportion (%) of cells with each intensity of staining calculated relative to the total number of cells in 5 fields were as follows: 0 (0–5%), 1 (6–30%), 2 (31–70%) or 3 (71–100%) (Fig. 1b). The nucleus ATF6-IHC score and cytosol ATF6-IHC score were each obtained by summing the multiplication of each proportion score by its respective intensity score, and the final ATF6-IHC score (potentially ranging from 0 to 14) was obtained by adding the nucleus score to the cytosol score (Fig. 1c).

#### IHC staining of p53

A streptavidin–biotin method was used for mutated p53 immunostaining. Antigen was retrieved from the tissues in pH 6.0 citrate buffer by autoclaving at 121 °C for 15 min. The endogenous peroxidase activity was quenched in the same way as for ATF6-IHC. The sections were incubated sequentially with polyclonal antihuman p53 antibody (1:50; DO-7, DAKO, Denmark) for 60 min at room temperature, MULTI for 30 min at room temperature, DAB for color development, 1% Mayer’s hematoxylin to counterstain, and dehydrated by immersion in a series of alcohols ending in xylene in the same way as ATF6 IHC.

As Shigaki et al. [23] reported, the p53 staining pattern of the nucleus was characterized as none, sporadic, mosaic, focal, and diffuse. Focal or diffuse patterns were considered as p53-IHC positive, regardless of the intensity. None, sporadic, and mosaic pattern were considered as negative for p53.

### Statistical analysis

Statistical analyses were carried out using SPSS (version 22.0, SPSS Inc, Chicago, IL. USA) software. For categorical data, the significance of between-group differences was estimated using the Wilcoxon signed-rank test and *χ* ^2^ test, as appropriate. For continuous variables, descriptive statistics of mean, median, and range were calculated, and the significance of between-group differences was estimated using the Mann–Whitney *U* test and Kruskal–Wallis test, as appropriate. A *p* value <0.05 was considered statistically significant.

### Ethical considerations

This study was conducted in accordance with the Declaration of Helsinki and its later amendments or comparable ethical standards. Written informed consent was obtained from all patients before enrollment, and the study protocol was approved by the Institutional Review Board of Tokyo Medical and Dental University.

## Results

### ATF6 expression in surgically resected CRC

**Fig. 2:**
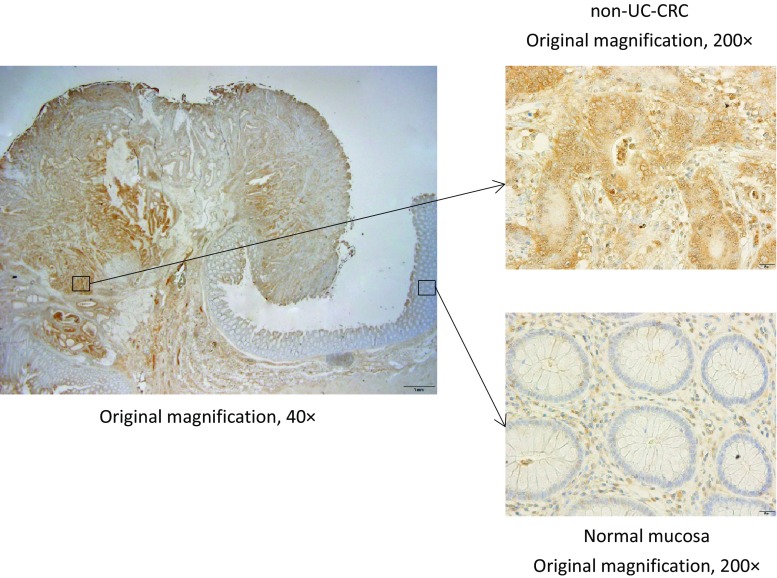
Representative ATF6 immunostaining in surgically resected non-UC-CRC and normal mucosa. *UC* ulcerative colitis, *CRC* colorectal cancer

Figure 2 shows representative results of ATF6-IHC staining in surgically resected CRC samples. While ATF6-IHC staining was negative in normal colonic epithelial cells (Fig. 2), it was positive in the cytoplasm and nucleus of the colon cancer cells. The median ATF6-IHC score of 137 surgically resected CRC specimens was 6.0 (range, 0–12). Table 1 shows the ATF6-IHC scores by major clinicopathological characteristics. There was no significant relation between ATF6-IHC score and any clinicopathological characteristics, except lymphovascular invasion (*p* = 0.026).

**Table 1:** ATF6-IHC score by characteristics of surgically resected CRCs

Characteristics	*n*	AFT6-IHC score: median (range)	*p*
Age (mean ± SD), years	66.1 ± 10.7		
Gender			
Male	75	5.0 (0–12)	0.983
Female	62	6.0 (2–11)	
CEA (ng/ml)			
≤5	98	5.0 (1–12)	0.722
>5	34	6.0 (0–12)	
CA19-9 (U/ml)			
≤37	119	6.0 (1–11)	0.417
>37	18	5.0 (0–12)	
Location of tumors			
Right sided	54	5.0 (1–11)	0.282
Left sided	83	6.0 (0–12)	
Histology (TNM-7th)			
G1	47	6.0 (1–12)	0.707
G2, 3	90	5.0 (0–11)	
Depth of tumor invasion (TNM-7th)			
T0	10	4.0 (1–8)	0.127*
T1	18	5.0 (3–10)	
T2	16	5.0 (3–11)	
T3	61	6.0 (0–12)	
T4	32	6.0 (3–10)	
Lymphovascular invasion			
Negative	28	5.0 (0–12)	0.026
Positive	108	6.0 (3–11)	
Lymph node metastasis (TNM-7th)			
Negative (N0)	72	5.0 (0–12)	0.081
Positive (N1, 2)	65	6.0 (3–11)	
Stage (TNM-7th)			
Stage 0	19	5.0 (1–8)	0.298*
Stage I	18	6.0 (3–11)	
Stage II	39	5.0 (0–12)	
Stage III	45	6.0 (3–11)	
Stage IV	16	6.0 (3–9)	

*CRC* colorectal cancer, *UC* ulcerative colitis, *SD* standard deviation

Mann–Whitney *U* test, *Kruskal–Wallis test

### ATF6 expression in endoscopically resected adenomas and pTis cancers

**Fig. 3:**
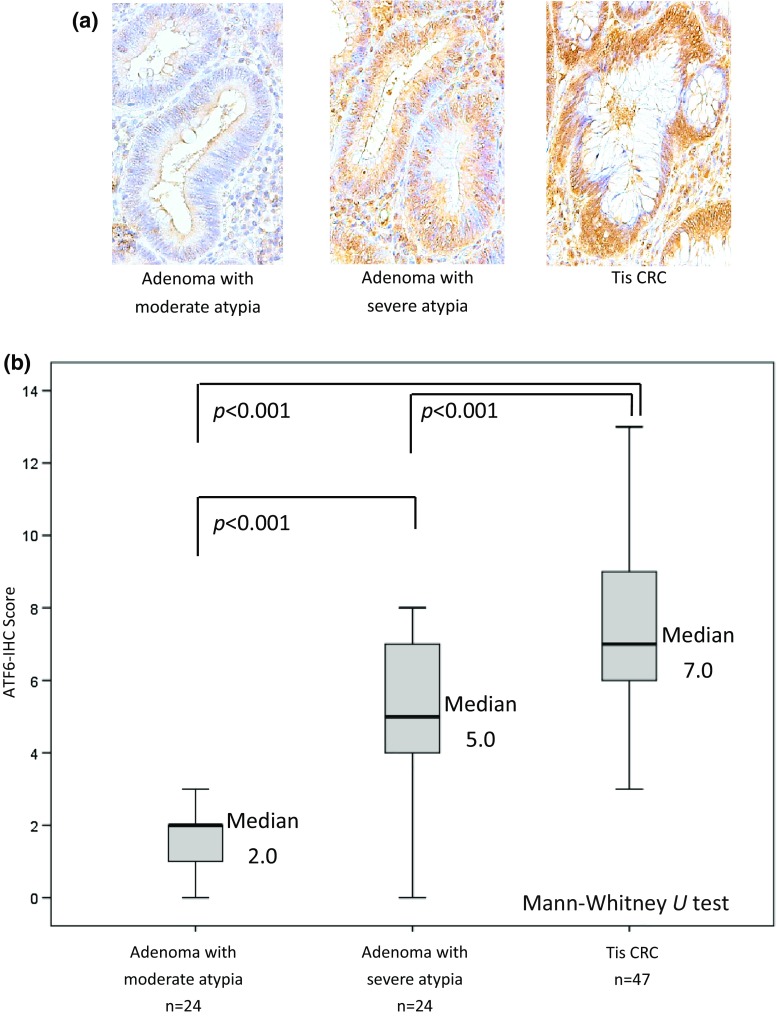
ATF6 expression in endoscopically resected adenomas and pTis cancers. **a** Representative ATF6 immunostaining in endoscopically resected samples. **b** ATF6-IHC scores of endoscopically resected samples by histological category. *CRC* colorectal cancer, * IHC * immunohistochemistry

Figure 3a shows representative results of ATF6-IHC staining in endoscopically resected adenomas and pTis CRCs, and Fig. 3b shows ATF6-IHC scores by lesion category. The median ATF6-IHC score of 95 endoscopically resected samples was 6.0 (range, 0–14). As atypia became more severe, the level of ATF6 expression increased; the median ATF6-IHC score was 2.0 (range, 0–4) in adenoma with moderate atypia, 5.0 (range, 0–8) in adenoma with severe atypia, and 7.0 (range, 3–14) in pTis cancer. There were significant differences among these three lesion categories (*p* < 0.001).

### ATF6 expression in UC samples

**Fig. 4:**
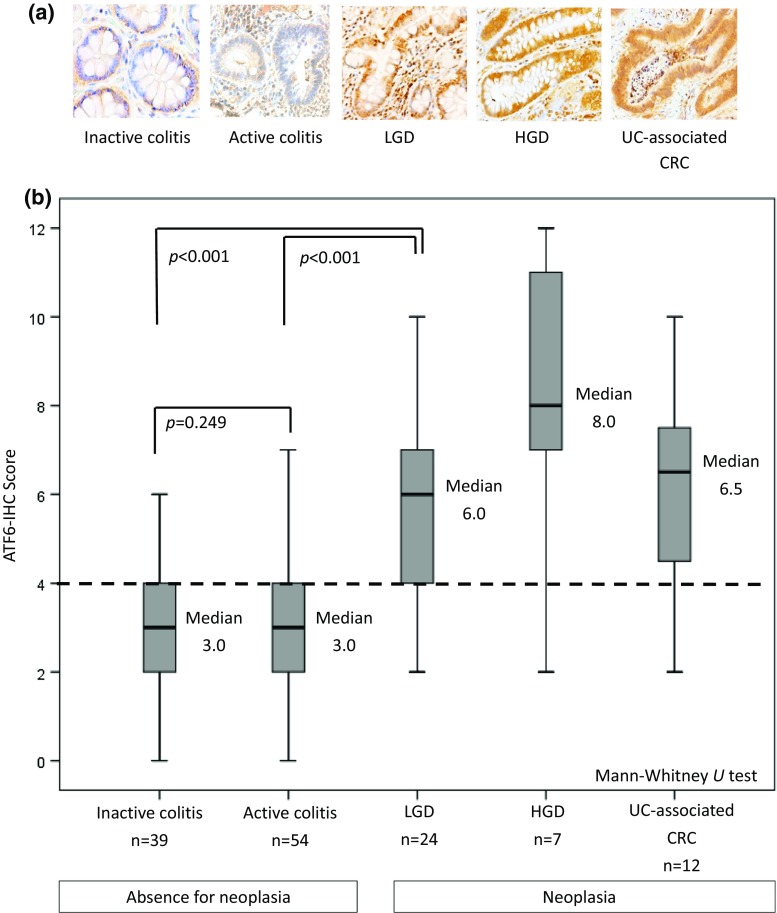
ATF6 expression in UC samples. **a** Representative ATF6 immunostaining in UC samples. **b** ATF6-IHC scores of UC samples by histological category. *UC* ulcerative colitis, *CRC* colorectal cancer, *LGD* low-grade dysplasia, *HGD* high-grade dysplasia, *IHC* immunohistochemistry

Representative results of ATF6-IHC staining in UC samples are shown in Fig. 4a, and Fig. 4b shows ATF6-IHC scores by histological category. The median ATF6-IHC score of 136 UC samples was 4.0 (range, 0–12). ATF6 staining was generally strong in the neoplastic lesions: the median ATF6-IHC score in 43 samples of neoplasia was 6.0 (range, 2–11), which was significantly higher (*p* < 0.001) than that in 93 samples without neoplasia (median, 3.0, range, 0–11). It should be noted that the ATF6-IHC score in LGD (median, 6.0, range, 2–12) was significantly different (*p* < 0.001) from the one in non-neoplastic lesions, i.e., inactive and active colitis samples (median, 3.0, range, 0–8, and median, 3.0, range, 0–11, respectively). No difference was observed between ATF6-IHC in inactive colitis and in active colitis (*p* = 0.249).

### The rate of ATF6-IHC and p53-IHC positivity in UC samples

**Fig. 5:**
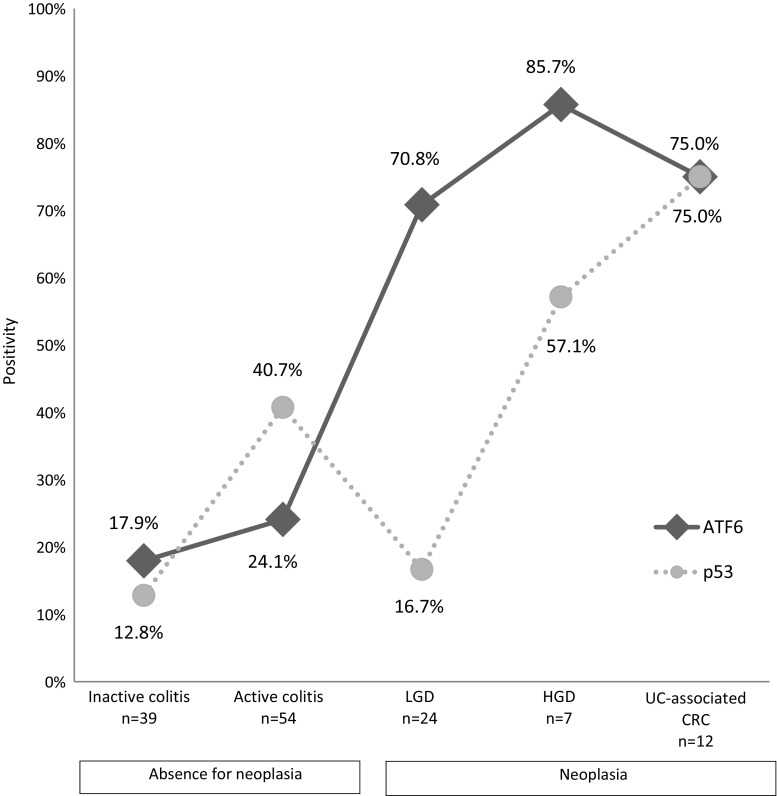
Rates of ATF6- and p53-IHC positivity by histologic status. *UC* ulcerative colitis, *CRC* colorectal cancer, *LGD* low-grade dysplasia, *HGD* high-grade dysplasia, *IHC* immunohistochemistry

Considering the median of ATF6-IHC score (4.0) of all 136 samples from UC patients and expression status by lesion category (Fig. 4b), we divided ATF6-IHC scores into two groups: ATF6-positive (ATF6-IHC score ≥ 5, *n* = 52) and ATF6-negative (ATF6-IHC score ≤ 4, *n* = 84). Status of p53-IHC was classified into p53-positive (*n* = 45) and p53-negative (*n* = 91) according to the method reported by Shigaki et al. [23]. The rates of ATF6- and p53-positivity in UC samples are shown in Fig. 5.

The rate of ATF6 positivity in neoplastic tissue was higher than that in non-neoplastic tissue. Notably, there was an obvious difference between the rate of ATF6-IHC positivity in LGD (70.8%) and that in inactive (17.9%, *p* < 0.001) and active colitis (24.1%, *p* < 0.001). On the other hand, there was no significant difference in the rate of p53-IHC positivity between LGD and lesions without neoplasia. While the rate of p53-IHC positivity exceeded 50% in HGD (57.1%) and UC-associated CRC (75.0%), that in LGD (16.7%) was similar to that in inactive colitis (12.8%) and lower than that in active colitis (40.7%) (Fig. 5).

### Accuracy of ATF6- and p53-IHC for diagnosing LGD

Using a total of 117 samples including LGD (*n* = 24) and non-neoplastic samples (*n* = 93), we evaluated the accuracy of ATF6-and p53-IHC for discriminating LGD from the inflammatory mucosa of UC. The sensitivity of ATF6-IHC was 70.8%, which was apparently higher than that of p53 (16.7%). The specificity of ATF6-IHC (78.5%) was relatively higher than that of p53 (71.0%) (Table 2). Totally, ATF6-IHC was more reliable than p53-IHC for a diagnosis of LGD.

**Table 2:** Accuracy of ATF6- and p53-IHC for diagnosis of LGD

	LGD	Absence for neoplasia	Sensitivity (%)	Specificity (%)
ATF6				
Positive	17	20	70.8	78.5
Negative	7	73		
p53				
Positive	4	27	16.7	71.0
Negative	20	66		

*LGD* low-grade dysplasia, *IHC* immunohistochemistry

## Discussion

ATF6, a UPR-related gene, is an ER-spanning transmembrane protein. Upon ER stress, ATF6 translocates to the Golgi apparatus, where it is cleaved to liberate an active, soluble transcription factor [12, 13, 16]. Through activating UPR, ATF6 may play an important role in promoting survival of dormant cancer cells via stimulating the mTOR pathway [15]. In addition, the ATF6 pathway is reported to be activated in inflamed colonic epithelium in UC patients [19, 24]. We therefore aimed to determine whether ATF6 expression correlates with the development and/or progression of CRC, especially UC-associated CRC, and whether ATF6 can be used for earlier diagnosis of dysplasia in UC patients.

In this study, we demonstrated that ATF6 protein was expressed in CRCs but not in normal colon epithelium. We also demonstrated that ATF6 expression increased with the increasing severity of atypia in adenoma, but had no effect on T, N, and M factors. Based on these results, we considered that ATF6 was elevated and detectable in lesions undergoing pre-cancerous atypical change in CRC development, which might be a very important phase of malignant conversion.

Regarding the results of the analysis of UC samples, ATF6 expression was low in non-neoplastic lesions, but significantly higher in LGD; the difference between non-neoplastic lesions and LGD was patently obvious. These findings showed that ATF6 expression was elevated in pre-cancerous lesions of UC-associated CRC as well as non-UC CRC. Accordingly, we hypothesized that ATF6 expression might be utilized as a supportive tool for detecting the early phase of neoplastic transformation in UC patients.

p53 mutation is an event in the carcinogenesis of various malignancies. p53-IHC has been often used for diagnosis of neoplasia in UC specimens, but its reliability is still controversial [2]. Alteration of p53 expression has been observed in more than 50% of samples from UC patients without neoplasia [25, 26], and the concordance rate between p53-IHC and p53 gene alteration detected by PCR single-stranded conformation polymorphism analysis is reported to be low [27]. Therefore, p53-IHC was reported to have insufficient accuracy and only approximately 11–40% sensitivity in the previous reports [28, 29].

In our results, p53-IHC could not distinguish the LGD and non-neoplastic mucosa, whereas rate of p53-IHC positivity was relatively high in HGD and cancer. The use of p53-IHC for diagnosis of LGD resulted in a considerable number of overlooked cases. Unequivocally, we considered that ATF6-IHC was more reliable than conventional p53-IHC for detection of LGD in UC patients.

Detection of dysplasia is very important in the clinical management of UC patients. A recent systematic review showed that, among UC patients with pre-operatively diagnosed LGD, approximately 30% of patients had more advanced neoplastic lesions in their surgically resected specimens [30]. While the managing of LGD is still controversial, when LGD is detected, total colectomy or endoscopic complete resection followed by close follow-up by repeat colonoscopy are recommended in the European and the Japanese guidelines [31–33]. We now expect that ATF6-IHC can be applied to biopsy samples and will have a great clinical impact if LGD can be diagnosed with sufficient accuracy using endoscopically obtained biopsy samples.

However, our study had some limitations. From our results, ATF6-IHC could not distinguish LGD from HGD, and could not predict whether the lesion become cancerous or invasive. If we can predict whether a detected lesion transform into more malignant one, it could be great help for planning the treatment. A future prospective study including a large number of endoscopic biopsy samples through long-term surveillance with endoscopy is needed. In the more distant future, the UPR system itself, including the ATF6 pathway, might be used as a target of treatment for UC and colorectal malignancies.

## Conclusions

Expression of ATF6, a UPR-related gene, was detected in lesions undergoing pre-cancerous atypical change in both non-UC and UC-associated CRC. ATF6 may help distinguish LGD from inflammatory regenerative epithelium in UC patients.

## Electronic supplementary material

Below is the link to the electronic supplementary material.

Supplementary material 1 (DOCX 158 kb)
