# Supplementary figures and images for: Expression of ATF6 as a marker of pre-cancerous atypical change in ulcerative colitis-associated colorectal cancer: a potential role in the management of dysplasia
Source: J Gastroenterol. 2017 Sep 7;53(5):631–41. doi: 10.1007/s00535-017-1387-1 (PMC5910497; doi:10.1007/s00535-017-1387-1)

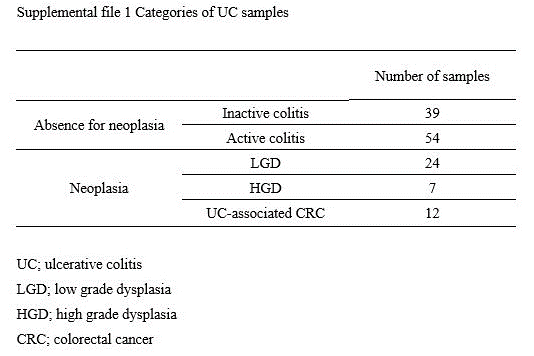


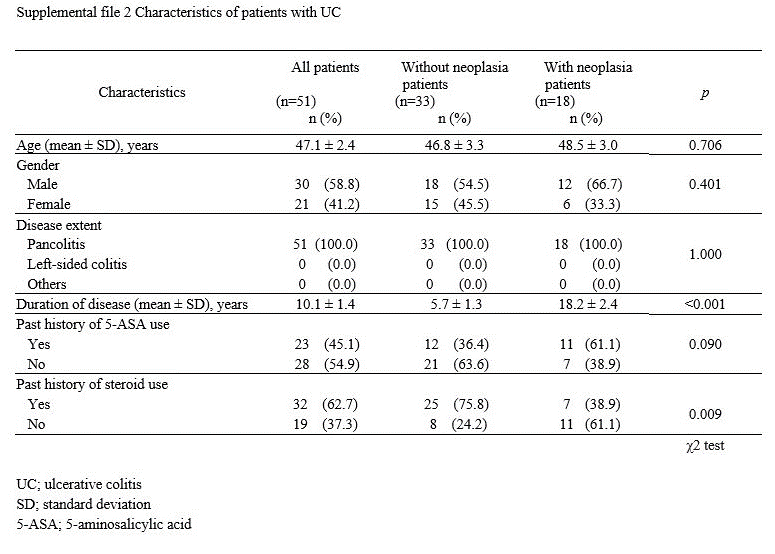


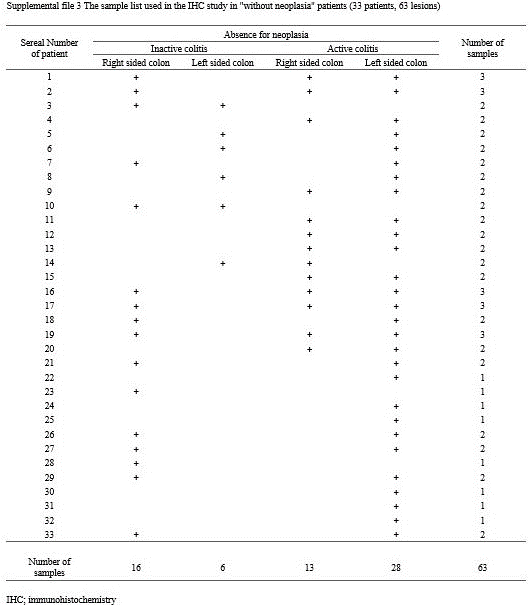


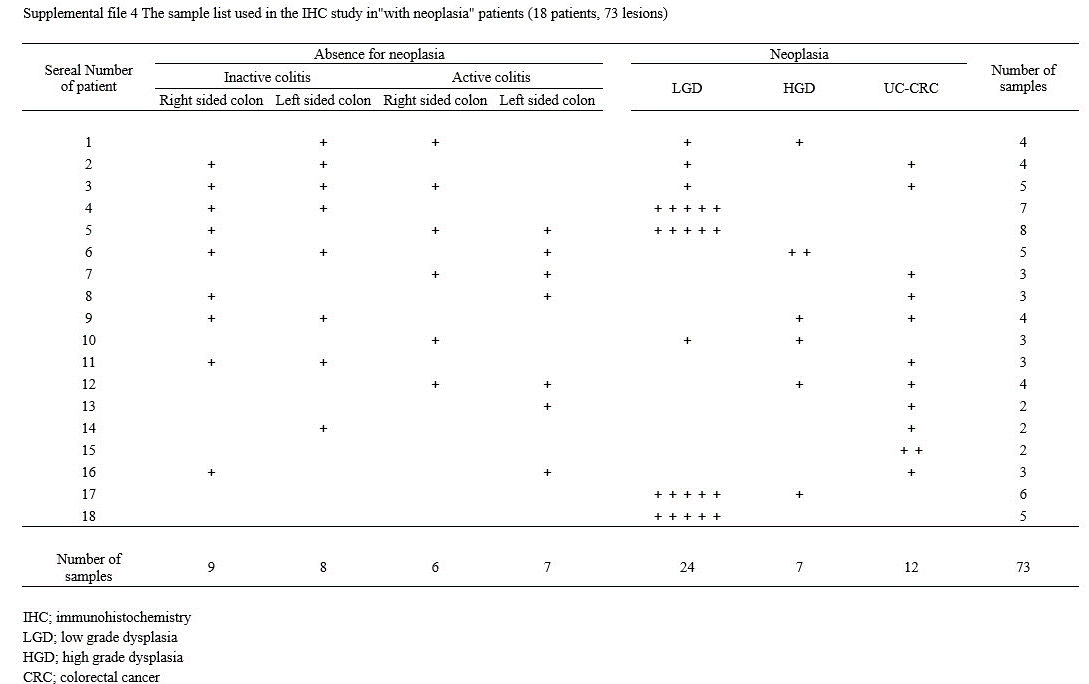

Supplement: Supplementary file 1 — Supplementary material 1 (DOCX 158 kb) [file 535_2017_1387_MOESM1_ESM.docx]
